# Tailoring Immune Responses toward Autoimmunity: Transcriptional Regulators That Drive the Creation and Collusion of Autoreactive Lymphocytes

**DOI:** 10.3389/fimmu.2018.00482

**Published:** 2018-03-08

**Authors:** Kim L. Good-Jacobson, Joanna R. Groom

**Affiliations:** ^1^Department of Biochemistry and Molecular Biology, Monash University, Clayton, VIC, Australia; ^2^Infection and Immunity Program, Biomedicine Discovery Institute, Monash University, Clayton, VIC, Australia; ^3^Walter and Eliza Hall Institute of Medical Research, Parkville, VIC, Australia; ^4^Department of Medical Biology, University of Melbourne, Parkville, VIC, Australia

**Keywords:** autoreactive B cells, germinal centers, transcription factors, Bcl-6, T-bet, interferon-gamma

## Abstract

T-dependent humoral immune responses to infection involve a collaboration between B and CD4 T cell activation, migration, and co-stimulation, thereby culminating in the formation of germinal centers (GCs) and eventual differentiation into memory cells and long-lived plasma cells (PCs). CD4 T cell-derived signals drive the formation of a tailored B cell response. Downstream of these signals are transcriptional regulators that are the critical enactors of immune cell programs. In particular, a core group of transcription factors regulate both B and T cell differentiation, identity, and function. The timing and expression levels of these transcription factors are tightly controlled, with dysregulated expression correlated to immune cell dysfunction in autoimmunity and lymphomagenesis. Recent studies have significantly advanced our understanding of both extrinsic and intrinsic regulators of autoreactive B cells and antibody-secreting PCs in systemic lupus erythematosus, rheumatoid arthritis, and other autoimmune conditions. Yet, there are still gaps in our understanding of the causative role these regulators play, as well as the link between lymphoid responses and peripheral damage. This review will focus on the genesis of immunopathogenic CD4 helper and GC B cells. In particular, we will detail the transcriptional regulation of cytokine and chemokine receptor signaling during the pathogenesis of GC-derived autoimmune conditions in both murine models and human patients.

## Introduction: Entry Points on the Path toward Autoreactive Antibody Production

Effective humoral immune responses depend on the ability to form and expand a population of B cells with high affinity for the foreign antigen. Both B cells and T helper cells employ a number of mechanisms to produce effector cells that help clear the antigen and form specialized immune memory cells. Yet, it is also the deployment of these mechanisms that put lymphocytes at risk of creating and expanding autoreactive cells that attack the host rather than the foreign invader and can lead to lifelong chronic disease.

Production of autoreactive B cells can occur at multiple stages of B cell development or differentiation. B cells that develop in the bone marrow, as well as those that differentiate in the periphery, undergo a number of checkpoints to exclude autoreactive cells from the immune repertoire. In particular, cells that produce an autoreactive B cell receptor will undergo deletion, receptor editing, or will be made anergic, such that autoreactive cells cannot participate in an immune response [reviewed in detail by Ref. (1)]. After development in the bone marrow, B cells migrate to the secondary lymphoid organs and undergo the final stages of maturation. Together, these developmental checkpoints result in a mature naïve B cell repertoire that is ready to respond to virtually any foreign antigen without the risk of cells that are specific to self-antigens mounting an attack. It is when these checkpoints fail (2) or are subverted by excessive extrinsic signals (3) that autoreactivity ensues.

Another major risk for generating newly autoreactive cells arises during an immune response. During a T-dependent response, activated antigen-specific B cells can either form an early wave of plasmablasts, which are low affinity for the antigen and mainly IgM, or they can form transient sites of proliferating cells to fine-tune the affinity of their receptor to antigen. This major site for affinity maturation is the germinal center (GC). GCs are transient sites formed within secondary lymphoid tissue from which high-affinity memory B cells and plasma cells (PCs) emerge. GC B cells activate the enzyme activation-induced cytidine deaminase (AID) which permits somatic hypermutation (SHM), a process in which random mutations are introduced into the B cell receptor. This is followed by selection and survival of high-affinity clones mediated by follicular dendritic cells (FDCs) and T follicular helper cells (Tfh). As mutations are random, SHM may result in a number of different outcomes for the antigen receptor, which can range from helpful to detrimental. Ideally, SHM will increase the affinity of the receptor for antigen. However, resulting clones may also no longer be specific, or have lower affinity for the antigen. Finally, mutated receptors may detect self-antigen. If left unchecked, these cells may result in the production of autoreactive antibody-secreting cells.

Transcription factors are molecular regulators that can activate or repress programs of gene expressions. They have critical roles in regulating cellular behavior during immune responses, including proliferation, differentiation, and migration of cells in response to the microenvironment. Both B and T cells rely on cytokines, chemokines, and other extrinsic signals to dictate their behavior throughout a response. Following these environmental cues, it is the molecular regulators downstream of these signals that orchestrate changes in gene expression and make functional and fate decisions. In particular, transcription factors regulate formation of the immunologic repertoire, as well as the differentiation of antigen-specific cells into effector and memory subsets during an immune response. These same extrinsic and intrinsic mechanisms that promote effective antibody responses and formation of immunity can also lead to autoimmunity. This review will focus on the points during an immune response at which B and Tfh cells can become dysregulated, and the underpinning transcription factors that balance appropriate responses to foreign pathogens with autoreactive cell formation. As such, we will focus on GC-derived autoimmune conditions, principally systemic lupus erythematosus (referred to within as lupus).

## Transcriptional Regulation of Class Bias in T and B Cells

**Figure 1:**
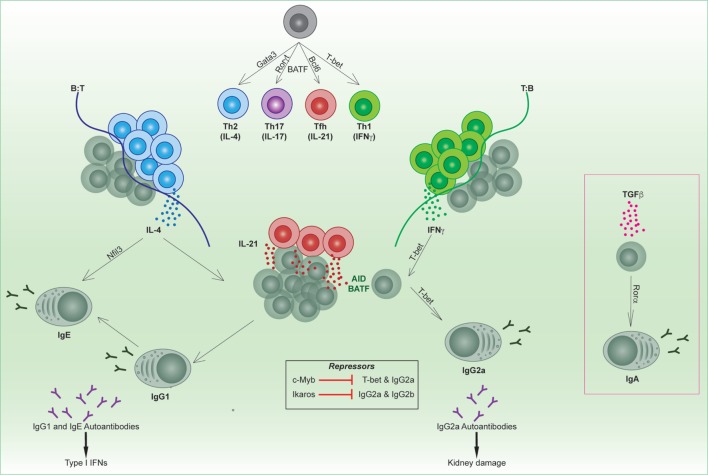
Transcriptional regulation of B and T follicular helper cell subsets. Unique transcription factors regulate the differentiation of T helper subsets, which in turn regulate the tailoring of the B cell response. Cytokines produced from specialized T helper subsets switch on transcription factors in activated B cells (e.g., T-bet, Rorα, Nfil3) that direct isotype switching to downstream immunoglobulin isotypes. Specific isotypes can mediate autoimmune conditions such as lupus.

The production of an effective humoral response relies on the coordinate orchestration of B and T cell behavior in unique areas of secondary lymphoid organs throughout the response. Depending on the pathogen type, such as a virus or bacteria, antigen-activated CD4 T cells will be skewed toward a Th1 (driven by T-bet), Th2 (driven by Gata3), or Th17 (driven by BATF and Rorγt) phenotype early in the response (Figure 1) (4). This results in secretion of specialized cytokines by these subsets that modulate the microenvironment and, in turn, direct B cell behavior.

B cells will tailor their B cell receptor to utilize the heavy chain with the effector function most suited to clearing the infecting pathogen. This is termed immunoglobulin (Ig) isotype switching, and is a process which relies on CD4 T cells. Cytokines produced from CD4 T cells, such as IL-4, IFNγ, or TGFβ, are able to direct Ig isotype switching, thus modulating the effector function of the antibody (5). AID expression and the transcription factor BATF are required for switching to all isotypes downstream of IgM (6–9). In addition, diversity in antibody isotypes is regulated by a small group of transcription factors that play context-specific roles in switching to the appropriate isotype in response to different cytokines (Figure 1). The most well characterized of these is the T-box transcription factor T-bet, which mediates production of murine IgG2a/c (referred to within as IgG2a) in response to IFNγ (10) as well as type I interferons (IFNs). By contrast, Nfil3 is required for IL-4-mediated induction of IgE (11), and Rorα in TGFβ-mediated induction of IgA (12). Furthermore, the transcription factor Ikaros can block the induction of murine IgG2a and IgG2b (13). It has not yet been determined whether other IgG subclasses (14), such as IgG1 or IgG3, have specific transcription factors that regulate their production.

Specific isotypes can also mediate immune disorders. For instance, mouse models of lupus are linked with excessive production of autoreactive IgG2a, which triggers antibody-dependent cell-mediated cytotoxicity. T-bet-mediated switching in response to IFN signaling is important not only for anti-viral humoral responses in mice (15, 16) but can also be immunopathogenic in murine models of lupus. T-bet is regulated by the transcription factor c-Myb in B cells, which represses the expression of T-bet during Th2 cell-biased responses (17). In both T and B cells, T-bet induces a number of gene expression changes that can affect cellular function and migration. For example, T-bet regulates CXCR3 expression and thus migration to sites of inflammation (18) and into kidneys of mice with lupus nephritis (19). In lupus-prone mice, IgG2a immune deposition is found on kidneys, in a similar fashion to antibody deposition in human patients.

There are also other isotypes that have been linked to autoimmunity, such as IgE. For instance, inactivation of the apoptotic mediators FAS/FASL causes autoimmune lymphoproliferative syndrome (ALPS) in humans and is also the basis of some murine lupus models. It was recently revealed that FAS inactivation in mice results in the production of “rogue” GC B cells, which gave rise to a high frequency of IgE-secreting PCs (20). Correspondingly, a subset of ALPS patients exhibits hyper IgE (20). Furthermore, lupus patients can also exhibit anti-DNA IgE antibodies. Self-reactive IgE antibodies may synergize with IgG autoantibodies to trigger type I IFN responses, correlating with disease severity (21). While the transcription factor Nfil3 is associated with induction of IgE, it is not clear whether inhibition of Nfil3 may ameliorate disease. In sum, it is clear that pathogens influence the class bias and hence effector function of both B and T cells. This has critical downstream consequences for humoral responses both in infectious responses and autoimmunity. Understanding the molecular regulation that drives the context-specific production or repression of different isotypes could potentially lead to new clinical targets for modulation in disease.

## The Interwoven Paths of B and CD4 T Cells During a Humoral Response

Positioning of B and CD4 T cells within different areas of secondary lymphoid organs regulates cellular interactions and exposure to signals within the microenvironment. The different migratory paths that B and T cells take during an immune response can dictate transcription factor expression, and determine the fate and function of these cells. In particular, a distinct T helper lineage, Tfh cells, is distinguished from other T helper cells subsets by its unique position within lymphoid organs, transcription factor expression (Bcl-6, c-Maf, BATF, IRF4, and Ascl2) (6, 7, 22–25), and cytokine production [predominantly IL-21, important for B cell proliferation, maintenance of GCs, and differentiation into antibody-secreting cells (26–29)]. The requirements for formation of these cells are imprinted *via* critical cellular interactions during the first few days of a humoral response (30–32), with DC–T cell interactions likely responsible for the initial upregulation of Bcl-6 within T cells (33). The expression of Bcl-6 regulates the gene encoding Ebi2 and is thus important for the convergence of T and B cells (34, 35). Bcl-6 expression is also important for determination of Tfh from Th1 *via* expression of Bcl6 over T-bet [reviewed recently in Ref. (18)]. However, it is important to note that in contrast to previous reports, T-bet can be co-expressed with Bcl-6 (36–38) during anti-viral responses. Furthermore, the absence of Bcl-6 does not automatically commit T helper cells to Th1 or other lineages (30). The ability of T cells to co-express Bcl-6 and T-bet has implications for the induction of autoreactive GCs, as detailed later in the review.

In the initial phase of a T-dependent immune response, activated antigen-specific B cells and CD4 T cells migrate to the border between B cell follicles and T cell areas. At the B:T border, B and T cells cooperate to promote each other’s differentiation into GC-precursor cells. This exchange of signals occurs both through direct cell surface ligand and receptor pairings, such as ICOSL–ICOS (32) and OX40L–OX40 (39, 40), as well as *via* SAP–SLAM signaling (41) and through T cell cytokine secretion. ICOS and OX40 have also been correlated to lupus pathogenesis in both humans and murine models (39, 40, 42). Tfh cells share this migratory path with other newly activated Th1 and Th2 effectors (43). Following Th1 cell-biased immunization, the ligands of CXCR3 are upregulated proximal to the B:T border and CXCR3-dependent migration into this area correlates with T cell-derived IFNγ production (44). Similarly, CXCR5^+^ Th2 cells also align to the B:T border following nematode infection (45). Combined, this work suggests that these early encounters adjacent to the B cell follicle expose antigen-specific B cells to CD4 effector cytokines. This cytokine microenvironment regulates the transcription factor programs that determine B and T cell fate to balance continued Bcl-6 (30–32, 46) upregulation and thus progression into GCs, or Blimp-1-induced PC differentiation or effector T cell differentiation.

B cells and early Tfh cells have two main paths from the B-T border: forming an extrafollicular plasmablast response or migrating into the follicles to form GCs. Autoreactive cells may be generated and/or expanded in either the extrafollicular response or the GC response. For an initial burst of protective antibody and/or in responses to bacteria such as *Salmonella enterica*, B cells may move to the extrafollicular areas of secondary lymphoid organs and differentiate into plasmablasts driven by transcription factors such as Blimp-1 and IRF4. Bcl6-expressing T helper cells help program extrafollicular responses both in response to T-dependent and T-independent antigens (46), as well in an autoreactive model (47), all of which is dependent on IL-21 (46–48). In addition to IL-21, a number of signals that are derived from T helper subsets, or produced by other cells present in secondary lymphoid organs are influential in selection and subsequent expansion of autoreactive clones. These include type I and type II IFNs (49, 50), toll-like receptor (TLR) signaling together with the survival cytokine BAFF (51–53), and other cytokines such as IL-6 and IL-17 [reviewed in Ref. (54)]. Generally, they act within secondary lymphoid organs, but some (e.g., IL-17) also act in peripheral inflamed organs, and some of these cytokines are produced in ectopic lymphoid organs (see section below).

B and T cells that do not go down this path instead migrate up through the interfollicular areas and into the follicle (31, 44). Ascl2 mediates chemokine receptor expression such that Tfh downregulate CCR7 and PSGL1 and upregulate CXCR5, which is required to migrate into the B cell follicle (24). Bcl-6 is further upregulated in the interfollicular regions (30, 31), finalizing commitment to the Tfh lineage. Within the follicle, B cells and Tfh collaborate within GCs to produce high-affinity memory B cells, long-lived PCs and memory Tfh cells (36, 55).

## GCs—A Site for B and T Cell Collusion

**Figure 2:**
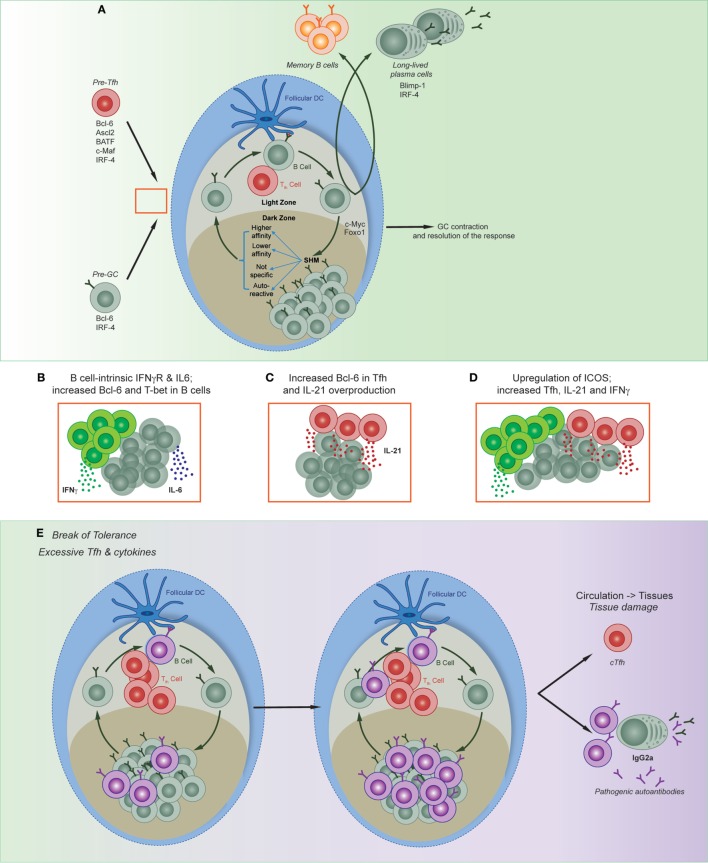
Regulation of multiple phases of the germinal center (GC) reaction. **(A)** Appropriate regulation of Tfh formation and function, GC affinity maturation, and resolution of the GC are all required to form immune memory. Red box denotes possible point of collusion between pre-Tfh and pre-GC B cells in autoimmune disease. **(B)** Both B cell-intrinsic and T cell-intrinsic mechanisms have been shown to contribute to the production and expansion of autoreactive clones; shown are three models described in text: **(B)** (49, 59, 60); **(C)** (48, 61); **(D)** (42, 62–65). **(E)** Disruption of both B and T cell pathways result in the production and expansion of autoreactive clones, and can lead to the migration of circulating T follicular helper cells, autoreactive B cells, and plasma cells that may result in tissue damage.

Germinal centers are specialized sites formed during immune responses that are responsible for the increase in affinity of B cells for the antigen (56–58). The three essential points of regulation of the GC response are: regulation of B cell behavior, regulation of Tfh, and resolution of the GC response itself (Figure 2). Dysregulation of any of these can lead to autoreactivity and/or the exacerbation of autoimmune disease.

Germinal centers are segregated into two zones—the dark and light zones—within which different functions occur. In the dark zone, B cells undergo proliferation and SHM of the B cell receptor. B cells will then migrate to the light zone, where they undergo selection *via* immune complexes on FDCs and compete for survival signals secreted by Tfh cells. Selected cells may then exit the GC and differentiate into memory B cells or long-lived PCs, or they will re-enter the dark zone to undergo another round of mutation and selection. T cell help of high-affinity GC B cells regulates cell cycle speed to mediate selection (56). This intricate process of cyclic migration between zones and interaction between different types of immune cells is important for appropriate regulation of affinity maturation. GC B cells have relaxed regulatory checkpoints within proliferating and mutating cells, and both clonal evolution (66) and the frequency of apoptotic cells (67) is similar between self-reactive clones and those specific to the immunizing antigen. Thus, once there is a break in tolerance to self-antigens, autoreactive clones can evade negative selection, undergo lymphoproliferation (68), with the consequential formation of B cell-mediated autoimmune conditions (69, 70). Dysregulation of T cell-intrinsic Bcl-6 (61) and overproduction of IL-21 by Tfh can further exacerbate disease (48, 54). The transcription factors Foxo1, BATF, and Myc mediate cycling between the light and dark zones, as well as selection of high-affinity cells (71–74). Whether dysregulation of these transcription factors within B cells enhance the conditions for selection and expansion of autoreactive cells remains largely uncharacterized.

## Regulatory Follicular T Cells and the Resolution of the GC

Foxp3-expressing follicular regulatory T cells (Tfr) are also important participants in GC responses (75). Their presence in the GC increases over time and they are thought to suppress Tfh numbers and function through molecules such as PD-1 (76). Chronic GCs increase the likelihood of generating autoreactive clones through epitope spreading (66, 68, 77); thus, the resolution of the GC response is essential to avoid standard immune responses against foreign pathogen from inducing autoimmunity. It is likely that Tfh, Tfr, and B cells all interact to shut down the GC; however, two Tfr-independent-related theories were recently put forward to explain the dissolution of the GC. The first was by McHeyzer-Williams and colleagues, who suggest that PCs directly interact with Tfh to downregulate Bcl-6 and IL-21 expression within Tfh (78). Supporting this, deletion of the PC transcription factor Blimp-1 specifically in B cells increased Tfh numbers (78). Furthermore, Toellner and colleagues demonstrated *in silico* that high-affinity antibodies feedback to mediate selection and to resolve the GC (79). Whether Tfr have a specific role in prohibiting autoreactive antibody formation is unclear, although there are two recent studies to suggest this possibility. Loss of Tfr through deletion of CD28 led to B cell-driven autoimmunity (80). Additionally, Ballesteros-Tato and colleagues recently demonstrated that IL-2-induced Blimp-1 suppression of Tfr resulted in an increase of anti-nuclear autoimmune antibodies after infection, by specifically promoting expansion of autoreactive antibody-secreting cells independent of GC and Tfh numbers (81). Furthermore, interactions between follicular T cell subsets and PCs through the inhibitory receptor PD-1 and its ligands may also suppress autoreactive GC cells (78, 82). Accordingly, PD-1 deficiency induces a lupus-like condition in mice (83) and PD-L1 deficiency can induce hyperactive Tfh responses in autoimmune arthritis (84).

## The Role of IFNγ and T-bet in the Creation and Collusion of Autoreactive GCs

Both type I and type II IFNs play important roles during the development of lupus (85), yet until recently it was unknown whether both were able to drive the formation of autoreactive GC in a B cell-intrinsic manner. Two recent publications tested the requirement of IFN signaling and the downstream molecular mechanism in B cell autoimmune models (49, 59). Although B cell-intrinsic type I IFN-accelerated lupus development, it was not absolutely required (49). By contrast, B cell expression of IFNγR, as well as BCR signals and either TLR or CD40L signals, induced Bcl6 and hence spontaneous GC formation (49, 59). Furthermore, B cell-derived IL-6 synergized with IFNγ to mediate autoimmunity (60). Interestingly, this process was specific to autoimmune GC development, as the combination of IFNγR, BCR, and either TLR or CD40L was not essential for the formation of GCs in response to foreign antigen.

While Domeier and colleagues identified a role for T-bet in the formation of spontaneous GCs (59), another study in the same issue determined that the B cell-intrinsic deletion of T-bet did not impact on GC formation (49). This latter study utilized the Wiskott Aldrich syndrome chimera model of autoimmunity. Wiskott Aldrich is an X-linked immunodeficiency caused by mutations in the *WAS* gene. Patients are prone to develop systemic autoimmunity, and mice that lack WAS protein is B cells establish autoimmune disease (86). While the authors found a critical mechanistic role for IFNγR in the formation of autoreactive GCs, this was not through the induction of T-bet (49). Yet, there are other studies demonstrating B cell-intrinsic roles of T-bet; in particular for the formation of the T-bet-expressing age-associated B cell (ABC) subset. Multiple groups have identified and characterized ABCs in murine autoimmune models as well as in elderly and autoimmune patients (87–89). While T-bet expression had been used as a marker of these cells, it was initially unclear whether they were causative of autoreactivity. Rubtsova and colleagues addressed this by conditionally deleting T-bet in mature B cells in lupus-prone mice, resulting in the amelioration of autoimmune disease (90).

While these studies demonstrated a B cell-intrinsic role for IFNγR, the role of IFNγR was T cell-intrinsic in the Roquin model of lupus (a model in which a mutation in the *roquin* gene results in an aberrant number of Tfh) (62, 63). Specifically, a lack of ICOS repression resulted in excess INFγ and IL-21, concomitant with a substantial induction of Tfh and consequently GCs (42, 62, 64). Deleting one allele of Bcl-6 ameliorated the autoimmune symptoms, thus demonstrating the dependency on Tfh for generating disease in this model (65). Together, the commonality of these models is the prominent role of IFNγ in generating autoreactive responses, and the parallel pathways B and T cells can take (depending on the model) to generate autoimmunity. Studies into the molecular mechanisms that may tip an IFNγ-mediated anti-viral immune response to one that promotes autoreactivity are needed. Finally, regardless of the cell-intrinsic nature of T-bet-induced autoreactivity, it would be beneficial to determine regulators of this pathway [such as c-Myb (17)] to identify new clinical targets for autoimmune patients. This is particularly important in the B cell lineage, as cells with similar characteristics to ABCs have now been described in a number of different contexts (91, 92), particularly those that still require effective interventions such as in chronic infectious diseases (87, 93) or autoimmune conditions (94–96).

## Transcriptional Regulation of Tfh-Derived Cytokines

The transcriptional regulation of Tfh cytokine production is of central concern to lupus pathogenesis, given the number of mechanisms described above in which excessive cytokine production by Tfh promotes autoreactivity. In particular, the members of the signal transducer and activator of transcription (STAT) family of transcription factors are critical regulators of Tfh-derived cytokines. A recent study investigated the transcriptional regulation of Tfh-derived cytokines in viral infection (97). In this setting, STAT4-dependent upregulation T-bet, in line with previous studies showing STAT4 promotes both Th1 and Tfh downstream of IL-12 signaling (37). STAT4 was required for both IFNγ and IL-21, presumably acting as an upstream inducer of both T-bet and Bcl-6 (37, 97). STAT3 and STAT1 are also important regulators of Tfh differentiation and function. Functional STAT3 deficiency in humans compromises the generation of Tfh and production of IL-21 (98), while T cells from patients with lupus display increased levels of total and phosphorylated STAT3 (99). STAT3 regulates the production of IL-21 downstream of IL-6, and a positive feedback loop exists between STAT4 and STAT3 to further promote IL-21 production (37). STAT3 also works together with STAT1 to promote Tfh differentiation, again through IL-6 induced Bcl-6 upregulation (100, 101). Interestingly, the reduction in Tfh differentiation observed in STAT3 deficiency is partially reversed with type I IFN blocking, which coincides with increased Bcl-6 expression (102). By contrast, STAT5 is induced in situations of high IL-2 to block Tfh differentiation in preference for Th1 *via* upregulation of Blimp-1 (103–105). Combined, the network of STAT transcription factors acts in concert with Bcl-6 and T-bet to specify key functional characteristics of Tfh which is relevant for lupus development.

## Migration to Sites of Immunopathology

While a lot of mechanistic insight has been gained by revealing the molecular factors underpinning B and T cell differentiation in secondary lymphoid organs, a lot less is known about whether these mechanisms also underpin local formation of autoreactive GCs in the tissues. Investigating human GC and Tfh dynamics and functionality is difficult as it relies on the attainment of tissues. As such, circulating Tfh (cTfh)—i.e., cells that possess some phenotypic and functional attributes of Tfh that are found in the blood—have been investigated as a proxy for what is occurring in the organs. It is currently unclear whether these cells are pre-Tfh or memory Tfh (106), as they do not express all markers of Tfh found in the tissues, notably Bcl-6. However, they have provided useful information about the clinical severity of diseases ranging from chronic infectious disease to autoimmune diseases. To that end, cTfh and associated serum cytokine levels such as IL-21 have been found to be elevated in patients with lupus and rheumatoid arthritis with active disease (107–112). Furthermore, a recent study also demonstrated that circulating Tfr were reduced in lupus patients, and that the ratio of Tfh/Tfr positively correlated with disease activity (113). As these cells do not express Bcl-6, other transcription factors may regulate their formation and/or migration. For instance, as previously noted, T-bet induces the expression of CXCR3, a chemokine receptor that is expressed by cTfh and is known to be important for directing cells to sites of inflammation. As cTfh do not express Bcl-6, it may be possible that cTfh are actually pre-Tfh that have been recruited into the blood before they fully differentiate in the follicle. Tfh-like cells in inflamed tissue from rheumatoid arthritis patients have been suggested to promote autoreactive plasmablast formation (114), but whether these cells originated from cTfh or were instead a non-Tfh subset produced locally (115) remains undetermined. Future work may resolve the following questions: (1) do cTfh cell phenotypes represent the cause or consequence of disease? (2) Have these cells been specifically recruited to sites of inflammation, or are they in the blood because they are dysfunctional and have simply been excluded from lymphoid organs?

## Chemokine Receptor Signaling That Regulates Migration of Immune Cells in Lupus

Lupus is a heterogeneous disease, in which the loss of varied tolerance checkpoints may result in similar disease phenotypes. Recently, transcriptional fingerprinting of patients has been highlighted as a means to deduce disease pathogenesis and stratify treatment protocols (116). This analysis has highlighted groups of patients with either primarily type I IFN or plasmablast signatures, suggesting a dichotomy of disease mechanisms. However, these patient phenotypes may be more intertwined. Several chemokines which may promote aberrant GC development are key IFN signature genes upregulated in lupus patients. Furthermore, the expression of the corresponding chemokine receptors has been correlated with B cells and/or PCs in autoimmune diseases, particularly lupus or rheumatoid arthritis. For example, elevated levels of serum CXCL10 (an interferon-inducible gene), CCL2, and CCL19 correlated with lupus activity (117–119). Furthermore, CCR6 has also been found to be upregulated on certain B cell subsets in lupus patients compared to healthy controls; however, the significance of this is currently unclear (120). The most well-characterized chemokine receptor in this context (regulation of cellular migration to sites of inflammation) is the T-bet-regulated CXCR3. CXCR3 mediates kidney disease in murine lupus nephritis (121, 122), and reduction of the transcription factor FLI1 results in amelioration of kidney disease in MRL/lpr mice with concomitant reduction in CXCR3^+^ T cells and CXCL9/10 expression (123). Yet, the role of these chemokine families in directly facilitating the development of either self-reactive GC in secondary lymphoid organs or ectopic GC structures in inflamed tissues (124), or in mediating tissue damage by GC-independent mechanisms, remains to be determined.

## Concluding Remarks and Future Perspectives

It is clear that dysregulation of either B cells or Tfh cells can result in the production of autoreactive GCs and antibody-secreting cells. Unchecked proliferation of Tfh and excessive production of cytokines, such as IL-21 and IFNγ, can collude with B cell-intrinsic mechanisms to induce autoimmune responses. Furthermore, transcription factors common to both B and T cells, such as Bcl-6, T-bet, and Blimp-1, can be hijacked to assist in driving aberrant responses to infection that lead to the formation and migration of autoreactive clones. In particular, T-bet, and the T-bet-regulated gene CXCR3, appears to be key to controlling the relocation of cells from secondary lymphoid organs to other tissues where ectopic GCs and/or local autoreactive plasmablast formation can result. However, there are still a number of open questions relating to how these molecular networks may be dysregulated. Studies done in both B and T cells have demonstrated that the timing and level of expression of these transcription factors are important. For instance, the expression level of T-bet is critical in regulating T cell fate decisions, with high levels of T-bet favoring Th1 development over Tfh in the CD4 lineage, or effector CD8 over memory CD8 T cells. While a molecular mechanism underpinning graded expression has been put forth for CD8 T cells (125), whether there is a similar mechanism in other lineages that express T-bet is currently unknown. There is also evidence that gradient expression exists in B cells (17). Given that B cell-intrinsic T-bet has been postulated to be causative of autoimmune disease in at least two murine models, it will be critical to understand whether the level of expression of T-bet can be causative of B cell dysregulation independent of extrinsic factors. Finally, while there has been a focus on IFN and T-bet in murine models in recent years, how this translates to pathogenesis in humans, and whether there are other transcription factor networks that drive the migration of cTfh, autoreactive B cells, and formation of ectopic lymphoid structures, requires further research. Thus, understanding the factors underlying the genesis of autoreactive GCs, *via* dysregulation of factors in the microenvironment and/or dysregulation of transcription factor networks, will be important in generating new targets for clinical intervention.

## Author Contributions

All authors listed have made substantial, direct, and intellectual contribution to the work and approved it for publication.

## Conflict of Interest Statement

The authors declare that the research was conducted in the absence of any commercial or financial relationships that could be construed as a potential conflict of interest.

## Abbreviations

ABCs, age-associated B cells; cTfh, circulating T follicular helper cells; GC, germinal center; IFN, interferon; Ig, immunoglobulin; PC, plasma cell; SHM, somatic hypermutation; STAT, signal transducer and activator of transcription; Tfh, T follicular helper cells; Tfr, T follicular regulatory cells; YFP, yellow fluorescent protein.
